# Characterization of the inflammatory/immune-neuroendocrine-BDNF interplay during affective episodes and euthymia in bipolar disorder patients: in the search of a peripheral reliable and highly predictive biosignature

**DOI:** 10.1192/j.eurpsy.2023.840

**Published:** 2023-07-19

**Authors:** M. Di Vincenzo, M. Ferrandino, R. Toricco, B. Collacchi, C. Musillo, L. Giona, M. Samà, F. Cirulli, A. Fiorillo, G. Sampogna, M. Luciano, A. Berry

**Affiliations:** 1 Department of Psychiatry, University of Campania “Luigi Vanvitelli”, Naples; 2 Center for Behavioural Sciences and Mental Health, Istituto Superiore di Sanità, Rome, Italy

## Abstract

**Introduction:**

Bipolar disorder (BD) is a psychiatric disease whose heterogeneity in phenotypic manifestations and disease severity hampers the diagnosis and the achievement of adequate therapeutic management. Increased pro-inflammatory cytokines and cortisol levels (CORT) have been observed in BD patients that might affect brain plasticity by decreasing Brain-Derived-Neurotrophic Factor (BDNF) levels. However, BD etiopathological mechanisms are still largely unclear and little is known about the interaction among these biomarkers and affective episodes.

**Objectives:**

To assess changes in peripheral endocrine and inflammatory markers, CORT awakening response, BDNF and cytokines levels during an acute phase of the disease and during euthymia and to evaluate whether these changes might be exploited as a biosignature of the disease.

**Methods:**

The study will be carried out on BD patients aged 18-65 who will be recruited during affective episodes (depressive, manic/hypomanic phase). In addition, a control group of 40 healthy subjects, age- and sex-matched will be also enrolled. All assessments will be carried out at the time of recruitment and after 3 and 6 months. Blood samples will be collected to evaluate cytokines (IL-1, IL-2, IL-6, IL-10, TNF-alpha, IFN-gamma) and BDNF. Hypothalamic-pituitary-adrenal (HPA) axis response will be assessed by measuring salivary cortisol levels upon awakening (cortisol awakening response – CAR). The psychopathological assessment will include the use of MADRS, YMRS and HAM-A for the assessment of psychiatric symptoms; PSP and C-SSRS for the assessment of global functioning and suicidal risk; IPSS and SRRS for the assessment of stress levels; CIRS for the evaluation of physical comorbidities.

**Results:**

We expect that 1) changes in inflammatory markers can predict the onset of acute phases of BD; 2) to observe significant differences in the levels of pro-inflammatory cytokines, CORT and BDNF between BD patients (during euthymia) and control subjects.

**Conclusions:**

Using a longitudinal approach, we will be able to evaluate whether the presence of affective symptoms in the BD patient is correlated with fluctuations in the levels of pro-inflammatory cytokines and chemokines, salivary cortisol and BDNF. Furthermore, the enrolment of control subjects will allow to evaluate if the inflammatory state and the activation of the HPA axis are steadily elevated in BD patients.

**“Funded by: Bando Ricerca Indipendente ISS 2021-2023 to A. Berry project code ISS20-9286e4091f8e”**

**Disclosure of Interest:**

None Declared

